# Neonatal Fc Receptor Regulation of Lung Immunoglobulin and CD103^+^ Dendritic Cells Confers Transient Susceptibility to Tuberculosis

**DOI:** 10.1128/IAI.00533-16

**Published:** 2016-09-19

**Authors:** Alexis Vogelzang, Laura Lozza, Stephen T. Reece, Carolina Perdomo, Ulrike Zedler, Karin Hahnke, Dagmar Oberbeck-Mueller, Anca Dorhoi, Stefan H. E. Kaufmann

**Affiliations:** Max Planck Institute for Infection Biology, Berlin, Germany; Weill Cornell Medical College

## Abstract

The neonatal Fc receptor (FcRn) extends the systemic half-life of IgG antibodies by chaperoning bound Fc away from lysosomal degradation inside stromal and hematopoietic cells. FcRn also transports IgG across mucosal barriers into the lumen, and yet little is known about how FcRn modulates immunity in the lung during homeostasis or infection. We infected wild-type (WT) and FcRn-deficient (*fcgrt*^−/−^) mice with Pseudomonas aeruginosa or Mycobacterium tuberculosis to investigate whether recycling and transport of IgG via FcRn influences innate and adaptive immunity in the lung in response to bacterial infection. We found that FcRn expression maintains homeostatic IgG levels in lung and leads to preferential secretion of low-affinity IgG ligands into the lumen. *Fcgrt*^−/−^ animals exhibited no evidence of developmental impairment of innate immunity in the lung and were able to efficiently recruit neutrophils in a model of acute bacterial pneumonia. Although local humoral immunity in lung increased independently of the presence of FcRn during tuberculosis, there was nonetheless a strong impact of FcRn deficiency on local adaptive immunity. We show that the quantity and quality of IgG in airways, as well as the abundance of dendritic cells in the lung, are maintained by FcRn. FcRn ablation transiently enhanced local T cell immunity and neutrophil recruitment during tuberculosis, leading to a lower bacterial burden in lung. This novel understanding of tissue-specific modulation of mucosal IgG isotypes in the lung by FcRn sheds light on the role of mucosal IgG in immune responses in the lung during homeostasis and bacterial disease.

## INTRODUCTION

Secretory immunoglobulin A (IgA) and IgM are exported by a dedicated receptor to the mucosa, where they coat pathogens and nurture beneficial microbiota (1). Yet the idea of the immune predominance of IgA in mucosal secretions has been challenged by the identification of the neonatal Fc receptor (FcRn) at mucosal sites in adult mammals (2). FcRn binds to conserved elements of the Fc portion of IgG at low pH, transports IgG across polarized epithelial barriers, and chaperones IgG between endocytic and degradative compartments (3). Selective expression of FcRn in epithelium was also found to enable antigens bound to IgG to be retrieved from the lumen to initiate systemic immunity to Citrobacter rodentium (4). FcRn guides monomeric IgG to the endocytic excretion pathway, extending its serum half-life by preventing lysosomal degradation (5). Finally, intracellular trafficking of antibody complexes within antigen-presenting cells via FcRn can influence lymphocyte activation with respect to opsonized particles (6, 7). Despite FcRn being the primary means by which IgG is transported across epithelial barriers, conflicting reports in a variety of infection models have questioned whether FcRn is required for the protective action of mucosal IgG, implying immune roles at the mucosal interface beyond neutralization of pathogenic determinants (8–10).

Inhaled air passes constantly through the lungs, continually introducing particulate and microbial matter into the lower airways, where it interacts with secreted antibodies. Many of the studies cited above have shed light on the role of FcRn-mediated IgG regulation and transport specifically in the gastrointestinal tract. Knowledge of the role of FcRn in the lung remains limited, although expression has been identified in parenchyma and recruited immune cells (11, 12). Indeed, FcRn has been harnessed to provide inhaled Fc-fusion vaccines access to systemic immunity (13, 14). We are interested in how IgG interactions with FcRn may be involved in homeostatic immune development in the lung and also in defense against respiratory infections by two bacterial species that require orchestrated multicomponent immune responses. The first, Pseudomonas aeruginosa, is an opportunistic pathogen that rapidly recruits innate immune cells to the lung parenchyma and is sensitive to complement deposition (15). The second, Mycobacterium tuberculosis, is a slow-growing pulmonary pathogen that disseminates to peripheral organs. Exponential growth in the lung is arrested by the arrival of specific Th1 T cells in the lung approximately 2 to 3 weeks after infection which promote formation of granulomas composed of diverse myeloid and lymphoid cells (16). Antibodies are thought to play a limited protective role in natural M. tuberculosis infection despite strong local recruitment of B cells to granulomas (17, 18). By studying *fcgrt*^−/−^ mice during homeostasis and bacterial infection, we aimed to characterize specific immune roles for IgG at the lung interface with the external environment.

## MATERIALS AND METHODS

### Bacteria.

Mycobacterium bovis BCG SSI 1331 (American Type Culture Collection [ATCC]; no. 35733), VPM1002 (recombinant BCG [rBCG]), and M. tuberculosis H37Rv (ATCC; no. 27294) stock was prepared as described previously (19). To generate green fluorescent protein (GFP)-expressing M. tuberculosis, competent H37Rv was transformed with plasmid pGFM-11 by electroporation and selected with kanamycin (20). P. aeruginosa strain PAO1 (PAO1-DSM; German Collection for Microorganisms and Cell Cultures) was grown in Luria broth (LB) at 37°C to the late exponential/early stationary phase and then subcultured for 2 h at 37°C to the mid-exponential phase before use. For CFU enumeration of the two pathogens, serial dilutions were performed in phosphate-buffered saline–0.05% Tween 80 (PBST) and plated onto Middlebrook 7H11 agar or LB agar and incubated at 37°C for 3 to 4 weeks or 16 h, respectively.

### Experimental animals and manipulations.

*Fcgrt*^−/−^ and P25 transgenic mice were purchased from Jackson Laboratories (USA) and bred in-house alongside C57BL/6 controls. Female mice were 8 to 16 weeks of age at the beginning of the experiments and were matched for age and sex and distributed randomly between treatment groups. Mice were kept under specific-pathogen-free (SPF) conditions at the Max Planck Institute for Infection Biology in Berlin, Germany, and experiments were carried out according to institutional guidelines approved by the local ethics committee of the German authorities (Landesamtes für Gesundheit und Soziale). For vaccination studies, P. aeruginosa strain PAO1 was killed by incubation for 2 h in 1% paraformaldehyde at 37°C and then washed 3 times in phosphate-buffered saline (PBS). Killing was confirmed by plating this preparation, and then the solution was adjusted to deliver 1 × 10^7^ bacteria per 6 μl dose into both nostrils at days 0, 8, and 14. Mice were infected intratracheally (i.t.) 40 days after vaccination or sham PBS treatment with 4 × 10^6^ P. aeruginosa strain PAO1 cells under mild anesthesia. A Glas-Col inhalation exposure system was used for aerosol infection of mice with low-dose (100 CFU) M. tuberculosis, confirmed by plating lung homogenates 1 day after infection. For generation of bone marrow chimeras, mice were irradiated with 9 Gy and immune reconstituted with 1 × 10^6^ bone marrow cells the following day. Chimeric mice were offered drinking water containing 125 mg/liter enrofloxacin (Bayer) for 4 weeks and allowed to recover for 12 weeks.

### Sample preparation.

Bronchoalveolar lavage was performed with 500 μl PBS supplemented with protein inhibitors (Roche) for antibody analysis. For antibody and CFU analysis, lungs were homogenized in PBST and protein inhibitors using GentleMACS dissociator M tubes (Miltenyi Biotec). For flow cytometry, lungs were harvested from perfused animals and digested with collagenase (0.7 mg/ml collagenase IV [Sigma-Aldrich]–0.3 mg/ml collagenase D [Roche])–RPMI 1640 medium at 37°C in 5% CO_2_ for 30 min. Single-cell suspensions were prepared by mechanical dissociation through a 70-μm-pore-size nylon mesh using RPMI 1640 medium with 10% fetal calf serum (Gibco).

### Flow cytometry.

Single-cell suspensions were stained on ice with the following eBioscience antibodies: B220 (RA3-6B2), Siglec-F (1RNM44N), CD103 (2E7), CD11b (MI-70), CD11c (N418), CD8α (53-6.7), CD69 (H1-2F3), CD4 (RM4-5), CD3ε (145-2C11), γδ T cell receptor (γδTCR) (GL3), NK1.1 (PK136), CD44 (IM7), Ly6G (1A8), Ly6C (HK1.4), MHCII (M5), and XCR1 (ZET; BioLegend). Intracellular IgG2c was detected with polyclonal goat antiserum (Southern Biotech) using Cytofix/Cytoperm (Becton Dickinson). Analysis was performed on LSR II or FACSCanto II (Becton Dickinson) flow cytometers. Data were analyzed with FlowJo (TreeStar).

### Antibody and isotype measurements.

For quantification of total or M. tuberculosis-specific antibodies, Multisorb enzyme-linked immunosorbent assay (ELISA) plates (Nunc) were coated overnight with goat anti-mouse antibody (Chemicon) (2 μg/ml of anti-Ig heavy and light chain) or protein lysate prepared by sonicating log-phase H37Rv M. tuberculosis. Nonspecific binding was blocked with 3% bovine serum albumin (Gibco). Serial dilutions were detected at an optical density (OD) of 405 using alkaline phosphatase-conjugated isotype-specific secondary antibodies (Southern Biotech). The abundance of various isotypes was measured using Procarta Isotyping Panel 2 Multiplex (eBioscience), acquired, and analyzed by Bioplex (Bio-Rad).

### Immunohistochemistry.

Sections (3 μm thick) of formalin-fixed lung were deparaffinized, and antigen retrieval was performed in citrate buffer. Cell membranes were permeabilized with 1% saponin and then blocked with 10% goat serum (Gibco) and a biotin/avidin blocking preparation (Dako). Endogenous IgG2b was detected with biotinylated polyclonal goat anti-mouse antibody (Southern Biotech) followed by streptavidin-Cy2 (Dako), while treatment with polyclonal rabbit anti-human myeloperoxidase (Dako) was followed by goat anti-rabbit serum conjugated to Cy3 (Dako). DAPI (4′,6-diamidino-2-phenylindol) (Sigma) was used to identify cell nuclei.

### Statistical analysis.

PRISM (Graphpad) was used for statistical analysis. Data that did not pass the Shapiro-Wilk normality test were analyzed using the nonparametric Mann-Whitney test. Normally distributed data were compared using the two-way Student's *t* test or one-way analysis of variance (ANOVA) followed by Dunnett's posttest depending on whether data from one group or from two or more groups, respectively, were being compared.

## RESULTS

### FcRn regulates the quantity and quality of IgG in lung secretions.

**FIG 1:**
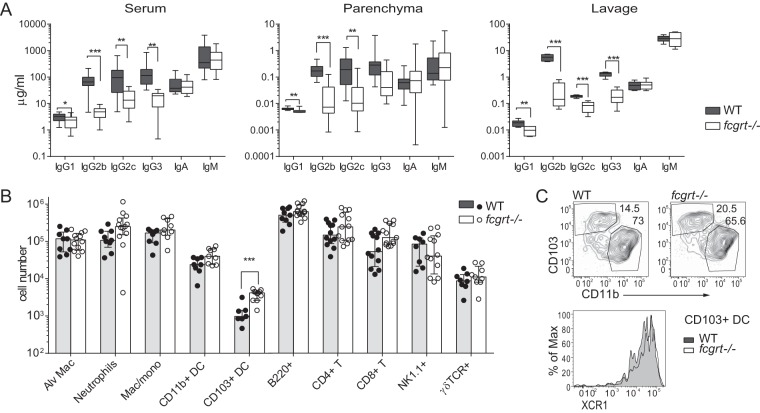
FcRn influences local immunoglobulin (Ig) homeostasis in lung, modulating resident CD103^+^ DC. (A) Relative levels of Ig isotypes in serum, homogenized lung tissue (Parenchyma), and bronchoalveolar lavage fluid (Lavage) measured by Bioplex. Box and whisker plots show the mean and total and interquartile ranges of cumulative data from 2 experiments where *n* = 10. Asterisks indicate *P* values (*, *P* < 0.05; **, *P* < 0.01; ***, *P* < 0.001). (B) Total numbers of innate and lymphoid immune cells analyzed by flow cytometry in lung. Data points show cumulative data from 3 experiments with the median and interquartile range. Alv Mac, alveolar macrophage; Mac/mono, macrophage/monocyte. (C) Representative flow cytometry data from the 2 experiments (*n* = 5), showing Ly6G-B220-CD11c^+^ MHCII^+^ DC from naive lung, as well as a histogram of XCR1 expression on overlaid WT and *fcgrt*^−/−^ CD103^+^ DC populations.

IgG from local and systemic immune responses is maintained in tissues and transported through the mucosa by binding to FcRn. We measured the relative abundances of antibody isotypes in serum, homogenized lung tissue, and bronchoalveolar lavage fluid (BALF) of wild-type (WT) and *fcgrt*^−/−^ mice to determine how ablation of IgG recycling or transport across lung epithelia influences homeostatic immunity in the lung. As previously described (3), *fcgrt*^−/−^ animals displayed reduced serum IgG levels, reflecting the well-described role of the FcRn extending the half-life of systemic IgG. This reduction in IgG isotypes was likewise observed in *fcgrt*^−/−^ lung parenchyma, implying that systemic IgG supplies tissues via diffusion from the circulation. Our analysis of antibody in BALF of WT and *fcrgt*^−/−^ mice revealed that, unlike IgM and IgA, luminal IgG strongly depended on FcRn expression, as *fcgrt*^−/−^ mice had 10- to 100-fold lower concentrations of locally dominant IgG isotypes. Intriguingly, the ratio of isotypes found in the airways of WT mice diverged from that observed in serum, such that IgG2b and IgG3 were the dominant antibody isotypes secreted into the mucosa. This discordant Ig isotype ratio depended on FcRn expression, as the levels of IgG isotypes in *fcgrt*^−/−^ lung more closely mirrored serum and tissue levels (Fig. 1A). Thus, FcRn in the lung qualitatively and quantitatively influences IgG in mucosal secretions.

### FcRn modifies homeostatic myeloid immune subsets in lung.

We performed immune phenotyping of lung-resident immune cells in WT and *fcgrt*^−/−^ animals to determine whether this receptor influences local leukocyte homeostasis. While most immune cell populations were comparable between strains, in the absence of FcRn we observed increased numbers of lung-resident XC chemokine receptor 1-positive (XCR1^+^) CD103^+^ dendritic cells (DC) compared to the results seen with WT animals (Fig. 1B and C). This correlated with increased homeostatic mRNA transcription of *itgae* (CD103) in lung, the DC growth factor *flt3*, and CD103^+^ DC-specific transcription factors *batf3* and *irf8* (see Fig. S1 in the supplemental material). However, expression of CD11b^+^ DC-associated transcription factor *irf4* was not influenced by FcRn expression (see Fig. S1). These data suggest that pulmonary IgG dampens local DC recruitment or differentiation during neonatal development or homeostasis. There was no evidence of ongoing inflammation in resting animals, as the two strains had similar numbers of local lymphocytes, and no differences were seen in the number or ratio of effector memory CD44^hi^ CD62^lo^ T cells in lung (see Fig. S2).

### FcRn is redundant for lung immunity to P. aeruginosa.

**FIG 2:**
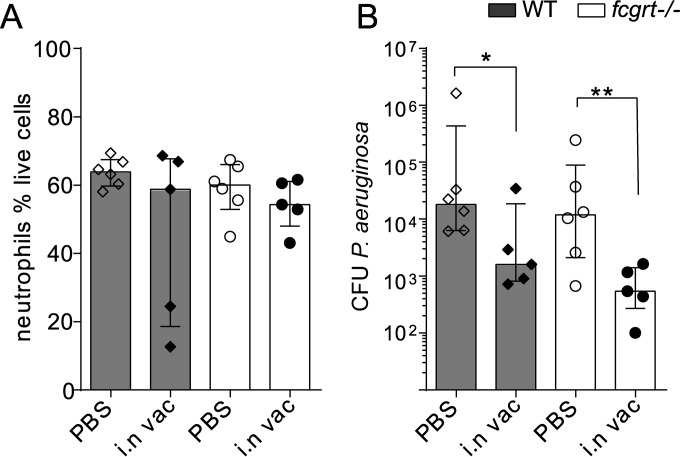
FcRn is not required for recruitment of neutrophils or bactericidal activity in P. aeruginosa infection. WT and *fcgrt*^−/−^ mice were vaccinated intranasally 3 times with paraformaldehyde-fixed P. aeruginosa. At 40 days postvaccination, vaccinated mice (i.n vac) and sham controls (PBS) were intratracheally infected with 4 × 10^6^ P. aeruginosa cells. (A) At 24 h postinfection, the percentage of lung-infiltrating neutrophils of live cells was measured by flow cytometry. (B) CFU of P. aeruginosa recovered from lungs 24 h postinfection. Data show medians and interquartile ranges of representative data from 2 experiments (*n* = 5 to 10 per group in each experiment). Asterisks indicate *P* values (*, *P* < 0.05; **, *P* < 0.01).

FcRn is expressed by myeloid cells and modulates lung DC populations and thus may influence local immunity to infection. In order to assess the role of luminal and intracellular IgG transport during acute bacterial infection requiring efficient recruitment of innate leukocytes, we first vaccinated WT and FcRn-deficient (*fcgrt*^−/−^) mice intranasally with formalin-killed P. aeruginosa to generate a pool of specific IgG in the lung as previously described (21). Vaccinated and sham-treated animals were challenged via intratracheal administration with virulent P. aeruginosa to determine whether local immunity was compromised in the absence of mucosal IgG and in the presence of lower levels of homeostatic IgG circulation in *fcgrt*^−/−^ animals. WT and *fcgrt*^−/−^ mutants were equally susceptible to acute bacterial pneumonia, suggesting that local IgG binding to FcRn is dispensable for the recruitment and antimicrobial action of neutrophils in a model where acute vascular leakage drives a rapid inflammatory response that is independent of adaptive immunity (Fig. 2) (22). Intriguingly, vaccinated *fcgrt*^−/−^ mice lacking mucosal IgG transport were protected from bacterial growth to the same extent as WT, suggesting that protective IgG acts within tissues or reaches the lumen due to paracellular antibody diffusion in P. aeruginosa infection (Fig. 2B).

### FcRn maintains intracellular stores of IgG in myeloid cells.

**FIG 3:**
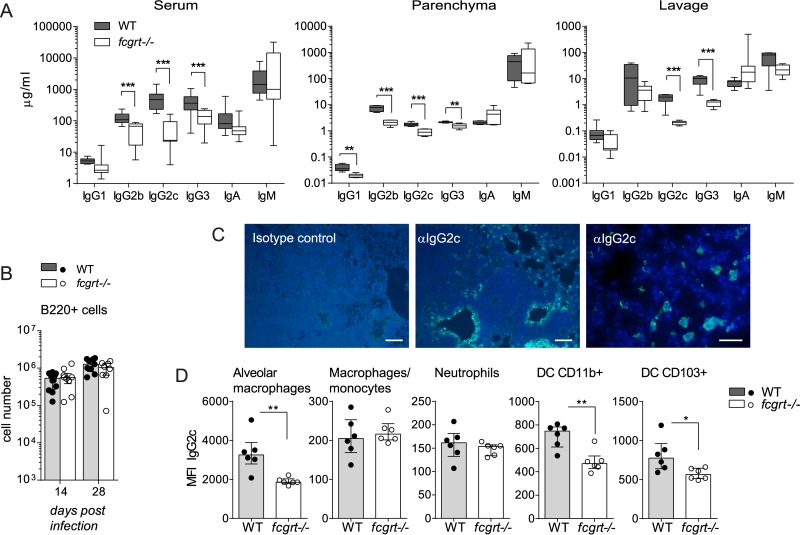
FcRn shapes the prevalence of IgG isotypes in lung after M. tuberculosis infection. WT and *fcgrt*^−/−^ animals were subjected to aerosol infection with M. tuberculosis. (A) Bioplex analysis of immunoglobulin isotypes on day 28 postinfection in serum, lung tissue homogenate (Parenchyma), or bronchoalveolar lavage fluid (Lavage). Box and whisker plots show the means and interquartile and total ranges, and asterisks indicate *P* values (*, *P* < 0.05; **, *P* < 0.01; ***, *P* < 0.001). (B) Flow cytometry quantification of B220^+^ lymphocytes in lung. Panels A and B show pooled data from 2 or 3 replicate experiments per time point (*n* = 5 to 6 animals per group in each experiment). (C) Representative results of immunohistochemistry of infected WT lung sections performed on day 28 after infection revealing intracellular IgG2c (green) in addition to nuclear DAPI staining (blue). Scale bars, 100 μm (left and middle panels) and 30 μm (far right panel). (D) Median fluorescence intensity (MFI) of intracellular IgG2c measured by flow cytometry in various lung-resident and infiltrating immune cells on day 28 postinfection. Data are representative of the results from 2 experiments (*n* = 4 to 6 animals per group in each experiment).

We determined the role of FcRn in a model of chronic bacterial infection where specific IgG levels and adaptive immunity develop gradually over the course of infection. WT and *fcgrt*^−/−^ mice were subjected to aerosol infection with M. tuberculosis, and the local and systemic antibody response was measured in serum, homogenized lung tissue, and BALF 28 days after infection. Although *fcgrt*^−/−^ mice exhibited reduced systemic and lung parenchymal IgG levels compared to WT mice, we observed similar 10- to 100-fold increases in the levels of IgG isotypes compared to their relative baseline levels, suggesting that a B cell response of equivalent magnitude had developed during tuberculosis (TB) (Fig. 3A). Indeed, local numbers of B cells in lung tissue increased equally in the two strains over the course of infection (Fig. 3B). IgG1 and IgG2b levels in BALF from infected *fcgrt*^−/−^ mice approached WT levels, indicating that the local antimycobacterial humoral immune response compensated for the reduced half-life of IgG in the absence of FcRn-mediated rescue from lysosomal degradation. However, levels of IgG2c and IgG3 in BALF from WT mice increased 10-fold over the baseline levels in an FcRn-dependent manner, suggesting that these isotypes require active export to the lung lumen across a functional epithelial barrier during TB (Fig. 3A). Immunofluorescence staining of infected WT lung tissue indicated that IgG was concentrated within bronchial epithelial cells but also within infiltrating immune cells (Fig. 3C). Intracellular reservoirs of IgG2c examined by flow cytometry showed that FcRn expression maintained depots inside resident alveolar macrophages and DC but not inside infiltrating neutrophils, macrophages, and monocytes during infection (Fig. 3D).

### FcRn modifies cell recruitment to granulomas during TB.

**FIG 4:**
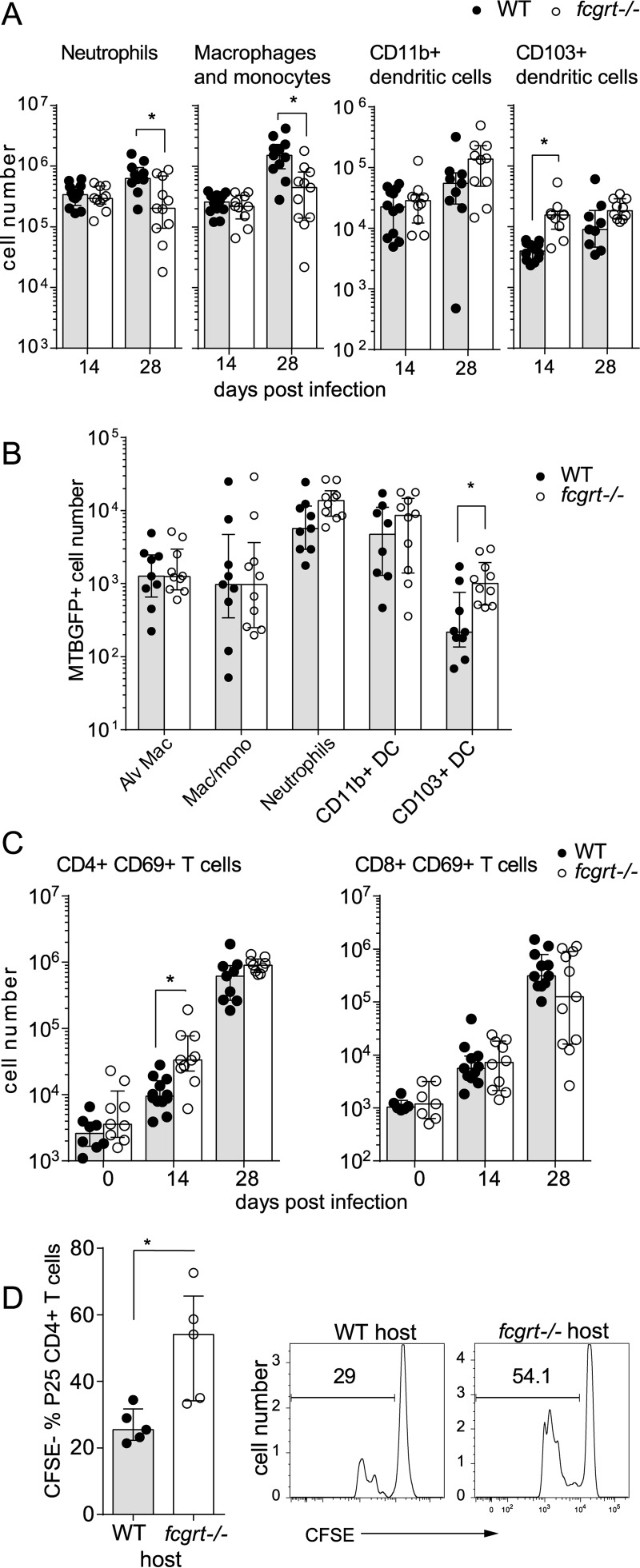
FcRn regulation of IgG during tuberculosis favors infiltration of myeloid cells and restricts T cell priming. Leukocyte infiltration in WT and *fcgrt*^−/−^ lung was analyzed by flow cytometry after aerosol infection with M. tuberculosis. (A) Quantification of the indicated myeloid cell subsets postinfection. (B) Quantification of the number of innate immune cells in the lung that were positive for GFP 28 days after infection with M. tuberculosis expressing GFP (MTBGFP+) compared to the number in the lung of control animals infected with nonfluorescent H37Rv. (C) Quantification of the number of CD44^hi^ CD69^+^-expressing CD3^+^ CD4^+^ or CD3^+^ CD8^+^ T cells in lung at the indicated time points before and after infection. (D) A total of 1 × 10^6^ transgenic TCR P25 CD4 T cells stained with carboxyfluorescein succinimidyl ester (CFSE) were intravenously transferred into M. tuberculosis-infected WT and *fcgrt*^−/−^ mice on day 12 following infection. On day 15 postinfection, the proportion of transferred T cells that underwent cell division was measured as shown on the representative flow cytometry histograms. All graphs show the median and interquartile range and cumulative individual data from 2 pooled experiments where *n* = 5 animals per experiment, except panel D, which shows results from one experiment of two performed. Asterisks indicate the *P* value (*, *P* < 0.05).

In order to investigate whether global FcRn expression affects the kinetics of leukocytes infiltrating the lung during TB, we performed flow cytometric analysis of M. tuberculosis-infected WT and *fcgrt*^−/−^ lung tissue. At the early day 14 time point, migratory innate immune cells such as neutrophils, monocytes, and macrophages as well as resident alveolar macrophages were numerically similar in the presence or absence of FcRn. In contrast, discrepancies in the numbers and proportions of neutrophils, inflammatory monocytes, and macrophages developed between *fcgrt*^−/−^ and control animals 1 month postinfection, implying that the influx or survival of inflammatory myeloid cells declined over the course of infection (Fig. 4A). Professional antigen-presenting cells, particularly the enlarged CD103^+^ DC population, were also more frequent in *fcgrt*^−/−^ lung during infection (Fig. 4A). Infection with M. tuberculosis GFP revealed higher proportions and numbers of infected CD103^+^ DC, suggesting that opsonized M. tuberculosis interacts with FcRn to inhibit phagocytic or bactericidal capacity in this population (Fig. 4B; see also Fig. S3 in the supplemental material). We measured upregulation of CD69, an adhesion molecule that is upregulated following T cell receptor stimulation permitting ingress from circulation to tissues, on T cells as a proxy marker of T cell activation. The two murine genotypes displayed equivalent activated CD8^+^ T cell numbers, but the numbers of CD4^+^ T cells expressing CD69 were increased in infected *fcgrt*^−/−^ mice at day 14 postinfection (Fig. 4C). To verify the increased T cell immunogenicity of *fcgrt*^−/−^ lungs, purified transgenic T cell receptor CD4^+^ T cells specific for the mycobacterial antigen 85B (Ag85B) were transferred into WT and *fcgrt*^−/−^ recipients 12 days following infection with M. tuberculosis. At 72 h after the transfer, the *fcgrt*^−/−^ lung environment drove increased proliferation of transferred cells as measured by carboxyfluorescein succinimidyl ester (CFSE) dilution (Fig. 4D).

Gene expression analysis of selected gene transcripts over the course of infection showed that ongoing inflammation gradually inhibited *fcgrt* expression in WT lung tissues. Numerically increased CD103^+^ DC and the corresponding influx of activated CD4^+^ T cells in *fcgrt*^−/−^ mice correlated with increased mRNA levels of lymphocyte chemoattractant transcripts such as *itgae*, encoding the protein CD103, and the lymphocyte- and DC-recruiting chemokines *cxcl19* and *ccl21* (see Fig. S1 in the supplemental material).

### FcRn deficiency leads to a transient decrease in M. tuberculosis load and pathology in lung.

**FIG 5:**
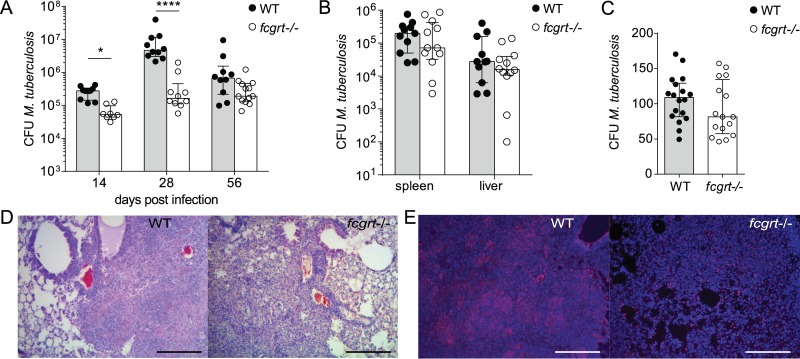
FcRn transiently affects disease progression in the lung during tuberculosis. WT and *fcgrt*^−/−^ mice were subjected to aerosol infection with M. tuberculosis. (A) CFU levels in lung were measured at various time points. (B) CFU levels in spleen and liver were measured at day 28 after infection. (C) Quantification of parenchymal M. tuberculosis was performed by plating total lung homogenates following bronchoalveolar lavage 1 day after infection. Graphs show cumulative data and the medians and interquartile ranges of the results of 2 experiments (*n* = 4 to 9 per group in each experiment). Asterisks indicate *P* values (*, *P* < 0.05; ****, *P* < 0.0001). (D and E) Representative histology sections of lung at day 28 postinfection showing the results of staining with hematoxylin and eosin to reveal immune infiltrated areas (D) and of immunofluorescent staining for myeloperoxidase (red) and cell nuclei (blue) (E). Scale bars, 1,000 μm.

The idea of the impact of FcRn on pulmonary immunity was supported by analysis of M. tuberculosis growth over the course of disease, which revealed that *fcgrt*^−/−^ animals were significantly protected from bacterial burden at 2 weeks and to a greater extent at 1 month after infection (Fig. 5A). Perhaps due to the ongoing suppression of *fcgrt* transcription during inflammation (see Fig. S1 in the supplemental material), this transient effect was lost 2 months postinfection (Fig. 5A). Peripheral organs of *fcgrt*^−/−^ animals were not similarly protected, suggesting that FcRn does not play a role in dissemination of M. tuberculosis (Fig. 5B). FcRn was also redundant for M. tuberculosis uptake by alveolar macrophages in the bronchoalveolar space following infection, as similar numbers of live M. tuberculosis could be retrieved from lavaged lung parenchyma from WT and *fcgrt*^−/−^ animals 1 day after aerosol administration (Fig. 5C). Histological analysis of lung tissue showed equivalent levels of dispersed cellular infiltration in *fcgrt*^−/−^ mice, despite the reduced M. tuberculosis burden in *fcgrt*^−/−^ animals (Fig. 5D). In addition to the reduced numbers of infiltrating neutrophils observed in *fcgrt*^−/−^ animals, the immunostaining pattern of myeloperoxidase was diminished within the lesions of *fcgrt*^−/−^ lungs, suggesting that early control of mycobacterial growth was associated with limited neutrophilic recruitment to granulomas (Fig. 5E). Thus, FcRn shapes the efficacy of the unfolding adaptive immune response to M. tuberculosis infection in the pulmonary mucosa.

### FcRn expressed by hematopoietic and stromal cells inhibits immunity in TB.

**FIG 6:**
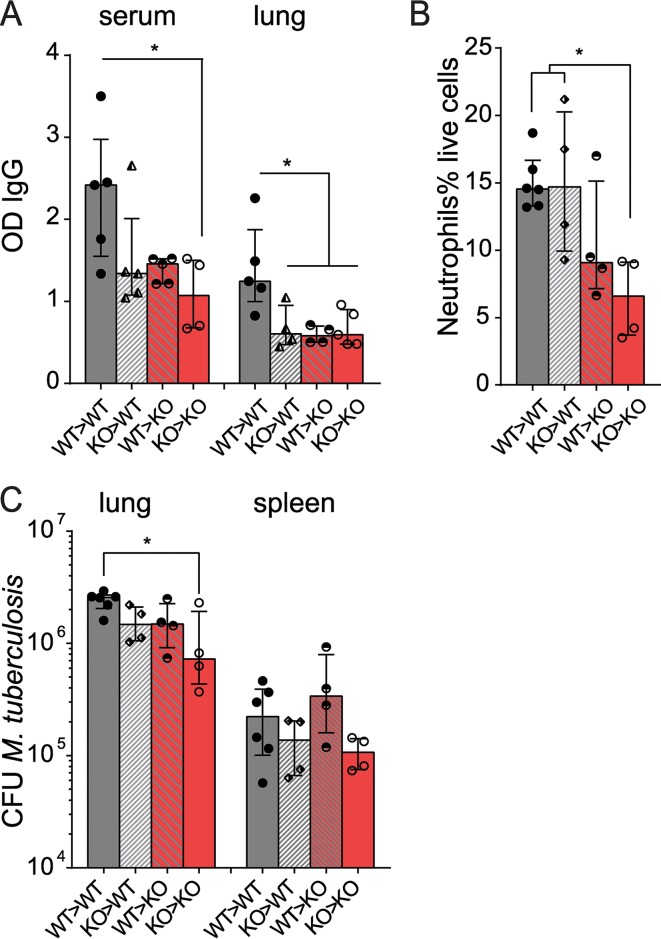
Stromal and immune cells expressing FcRn contribute to transient uncontrolled bacterial growth in tuberculosis. Lethally irradiated WT and *fcgrt*^−/−^ mice were reconstituted with WT or *fcgrt*^−/−^ bone marrow to create mixed or control chimeric mice, which were subjected to aerosol infection with M. tuberculosis and sacrificed 28 days later for analysis. (A) Specific IgG in serum and lung homogenate was quantified by ELISA. KO, knockout. (B) Ly6G^+^ CD11b^+^ neutrophils were measured as a proportion of live cells by flow cytometry. (C) Bacterial loads were quantified in the lung and spleen. Graphs show the medians and interquartile ranges of representative results from two experiments (*n* = 4 to 6 per group in each experiment). Asterisks indicate the *P* value (*, *P* < 0.05).

Last, we generated bone marrow chimeras to investigate whether IgG transport via FcRn to and from airways, or intracellular IgG trafficking via the FcRn within immune cells, correlated with susceptibility to tuberculosis. Both immune and nonimmune cells contributed to maintenance of inflammatory IgG levels in mixed bone marrow chimeras (Fig. 6A). However, the protective phenotype of fewer neutrophils and decreased M. tuberculosis burden of *fcgrt*^−/−^ mice at 1 month postinfection was recapitulated only when both hematopoietic and nonhematopoietic cells lacked FcRn (Fig. 6B and C). FcRn modulated immunity in both tissues and within mucosal secretions over the course of M. tuberculosis infection. Our results suggest that both functions of the FcRn, ongoing IgG transport across tissue barriers and within intracellular compartments, enhance pathology and inhibit bacterial killing during tuberculosis.

## DISCUSSION

This report reveals the nuanced balance between IgG recycling and transport by FcRn under homeostatic and inflammatory conditions in the lung. Analysis of the relative levels of FcRn-dependent transport of Ig subclasses in murine gut led to the proposal of the affinity hierarchy IgG2a/IgG2c = IgG1>IgG2b>IgG3, and the relative concentrations of IgG isotypes matched this descending order of FcRn binding (23). However, we observed increased levels of IgG2b and IgG3 compared to IgG2c in the lung, though these isotypes were present at similar concentrations in the serum and lung parenchyma. Thus, the IgG isotypes with the lowest affinity for FcRn are found at the highest concentration in lung secretions, leading counterintuitively to a divergence from serum isotype ratios in an inverse relationship to affinity for the receptor. A similar pattern was described in mammary secretions and healthy human lungs (24–26), and it has been suggested that high-affinity IgG1 and IgG2c antibodies locally saturate FcRn, resulting in preferential release of low-affinity IgG3 and IgG2b into the lumen. The prevalent IgG2b and IgG3 isotypes have relatively increased bactericidal and opsonizing properties (27), and perhaps their dominance represents an adaptation maintained by FcRn in order to maximize the sterilizing efficacy of mucosal antibody in the lung. However, the same processes could damage sensitive tissues during the persistent inflammation that characterizes tuberculosis. Parallels may be drawn between the detrimental actions of FcRn in this model and previous findings showing that it can promote IgG complex-mediated tissue pathology in autoimmune inflammatory disorders (28–30). Indeed, specific antagonists of FcRn are being pursued as novel therapeutic measures for this reason (31).

Our data from murine P. aeruginosa infection support the notion that FcRn-mediated IgG transport into the lumen becomes redundant under inflammatory conditions because paracellular transport and vascular leakage bypass the receptor (8, 10). Uncompromised immunity to P. aeruginosa infection in *fcgrt*^−/−^ lung and equivalent uptake of M. tuberculosis GFP by neutrophils suggest a limited cell-intrinsic role for FcRn in neutrophil function *in vivo* during acute infection. This contrasts with *in vitro* studies showing that FcRn is required for optimal granulocyte phagocytosis of IgG-coated, heat-killed Streptococcus pneumoniae (32), but perhaps other phagocytic receptors or pathogen-specific determinants can compensate for FcRn-Ig interactions *in vivo*. The slight difference in M. tuberculosis burdens in lung at day 14, before the arrival of CD4^+^ T cells and production of specific IgG, is therefore surprising and leaves room for the possibility that FcRn can influence intracellular bacterial replication or local production of bactericidal peptides.

Numerous studies have shown that the early recruitment of CD4^+^ T cells to the lung is the dominant factor determining bacterial control and lung pathology during tuberculosis (16, 33). Our studies join a growing body of literature showing that failed T cell responses result in excessive inflammation during tuberculosis in humans, primates, and mice, in association with increased bacterial load (34, 35). CD103^+^ DC have been observed to promote increased Th1 and Th17 CD4^+^ T cell responses *in vitro* compared to monocyte-derived CD11b^+^ DC, T helper subsets that have been shown to be particularly beneficial during TB (36, 37). It is tempting to speculate that the numerical increase of both infected and bystander CD103^+^ DC in *fcgrt*^−/−^ mice maintains improved T cell priming in tuberculosis. Perplexingly, FcRn has previously been reported to aid uptake and presentation of IgG-complexed antigen for CD4^+^ and CD8^+^ T cells by CD11b^+^ DC in several models (38, 39). Yet, during tuberculosis, we observed the inverse phenomenon of increased infection rates and T cell activation in the absence of the receptor at the early day 14 time point after infection, and, even when bacterial growth rates diverged between WT and *fcgrt*^−/−^ mice by day 28, similar levels of CD4^+^ T cell activation could be observed despite the lower antigenic burden in protected *fcgrt*^−/−^ animals. Perhaps DC in various tissue compartments have different requirements for the receptor, or perhaps the solubility of Ig antigen complexes and their interactions with FcRn differ between model systems. The striking increase in resident lung DC observed in *fcgrt*^−/−^ mice during homeostasis emphasizes that the role of FcRn during myeloid cell development in tissues should be targeted in future studies.

We observed that increased *flt3* transcript expression correlated with an expanded CD103^+^ DC population in *fcgrt*^−/−^ animals, supporting previous publications showing the systemic importance of this growth factor for DC development (40, 41). The explanation of how local IgG or commensal microbes may inhibit lung DC development or recruitment via FcRn remains elusive. Analysis of contrived commensal colonization of germfree *fcgrt*^−/−^ animals or acquisition of lung immunity in neonates will have to address this issue in the future. Interestingly, an elegant study by Koch et al. recently showed a strikingly similar phenomenon in neonatal gut, where maternal IgG2b and IgG3 in the mucosa suppressed downstream T cell priming to mucosal antigens (42). Our results confirm that local dendritic cells are influenced by the prevailing signals derived from mucosal IgG during development. Mucosal IgG may limit access of antigens to lung or draining lymph nodes, as was previously shown to be the case in the context of barrier integrity of the gut epithelium in neonates (43).

Our results clearly demonstrate an indirect role for IgG in modulating cell recruitment to granulomas. We have shown here that the quantity and quality of IgG in airways are maintained by FcRn, which negatively impacts homeostatic populations of DC in the lung and subsequent T cell immunity and neutrophil recruitment during tuberculosis. This novel understanding of tissue-specific modulation of mucosal IgG isotypes in the lung by FcRn sheds light on the role of mucosal IgG for immune responses in the lung during homeostasis and bacterial disease.

## Supplementary Material

Supplemental material
