# Supplementary material for: Neonatal Fc Receptor Regulation of Lung Immunoglobulin and CD103+ Dendritic Cells Confers Transient Susceptibility to Tuberculosis
Source: Infect Immun. 2016 Sep 19;84(10):2914–21. doi: 10.1128/IAI.00533-16 (PMC5038074; doi:10.1128/IAI.00533-16)
Supplement: Supplemental material [file supp_84_10_2914__index.html]

Neonatal Fc Receptor Regulation of Lung Immunoglobulin and CD103+ Dendritic Cells Confers Transient Susceptibility to Tuberculosis — Supplemental material 

# Neonatal Fc Receptor Regulation of Lung Immunoglobulin and CD103+ Dendritic Cells Confers Transient Susceptibility to Tuberculosis

## Supplemental material

- Supplemental file 1 -

  Fig. S1. Neonatal Fc receptor expression restricts transcription of mucosal DC-associated transcription and growth factors, cell markers, and chemokines in lung during homeostasis and disease. Fig. S2. Homeostatic T cell populations in naive mouse lung are independent of FcRn expression. Fig. S3. Intracellular niches of *M. tuberculosis* infection in lung.

  PDF, 128K
