# Supplementary material for: Neonatal Fc Receptor Regulation of Lung Immunoglobulin and CD103+ Dendritic Cells Confers Transient Susceptibility to Tuberculosis
Source: Infect Immun. 2016 Sep 19;84(10):2914–21. doi: 10.1128/IAI.00533-16 (PMC5038074; doi:10.1128/IAI.00533-16)
Supplement: Supplemental material [file IAI.00533-16_zii999091843so1.pdf]

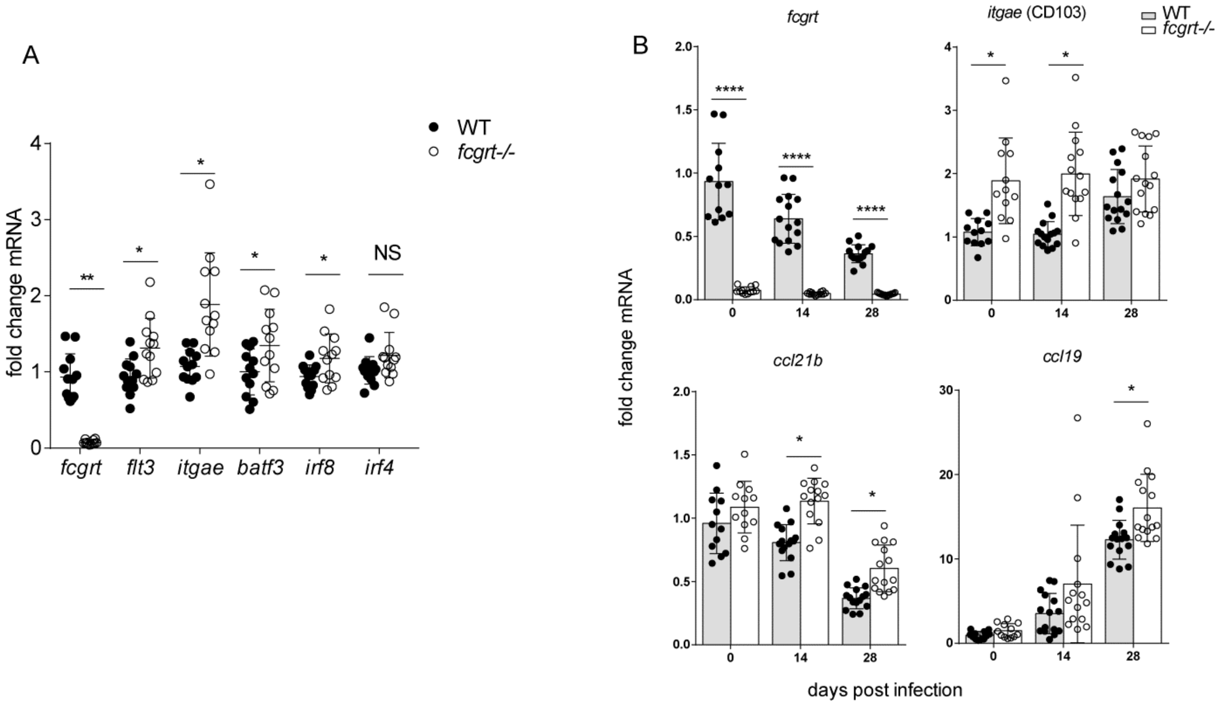

Supplementary figure 1. Neonatal Fc receptor expression restricts transcription of mucosal DC associated transcription and growth factors, cell markers and chemokines in lung during homeostasis and disease. mRNA was isolated from resting and *M. tuberculosis* infected lung tissue at the timepoints indicated. Relative transcript abundance was measured using Taqman probes (Applied Biosystems) and the 96.96 Dynamic Array Integrated Fluidics Circuit (Fluidigm). Transcripts were normalised to GAPDH expression for each sample, and the fold change (2-ΔΔCt) was calculated in reference to the mean expression of each gene in mRNA pooled from 10 naïve WT mice assayed in triplicate. A. Relative expression of dendritic cell development associated transcripts in naïve WT and *fcgrt-/-* mice. B Fluidigm analysis of mRNA transcripts of the indicated genes in lung before and after infection. Plots show the mean and standard deviations of pooled data from two independent experiments where n=12-16 for each timepoint in total. Statistical significance of various genes before infection (A) was calculated using student's T test, whereas changes over time (B) were determined using the Holm-Sidak method of multiple t-tests. *p<0.05, **<0.01.

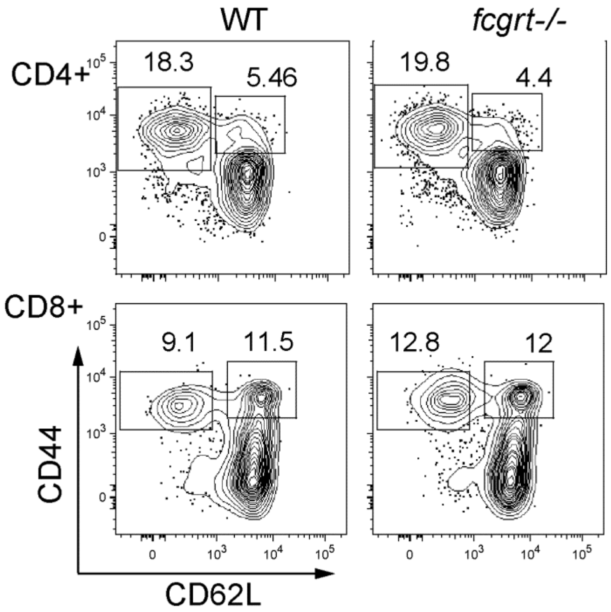

Supplementary figure 2. Homeostatic T cell populations in naïve mouse lung are independent of FcRn expression. Quantification of flow cytometry of T cell populations in naïve WT anf *fcgrt-/-* lungs gated on CD3+CD4+ or CD3+CD8+ T cells, and indicating the CD44hi CD62- T effector memory and CD44hi CD62+ T central memory subsets. Representative flow cytometry from 2 experiments where n=5

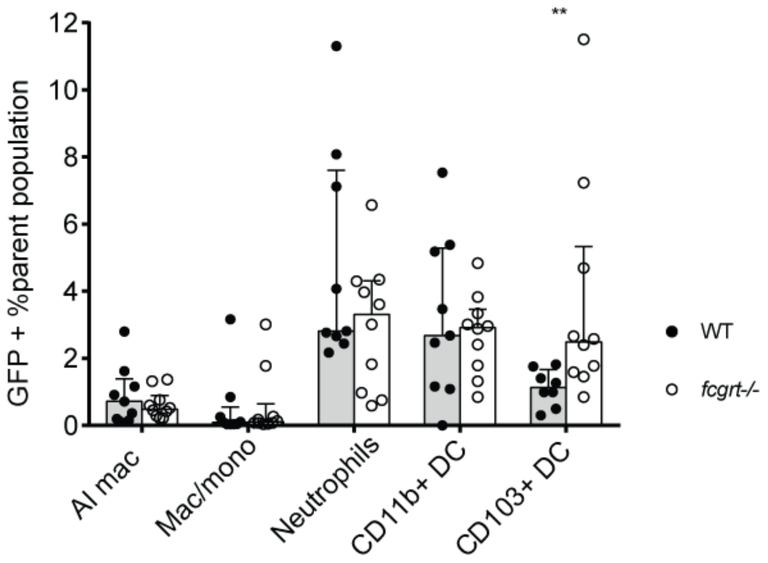

Supplementary figure 3. Intracellular niches of *M. tuberculosis* infection in lung. WT and *fcgrt-/-* mice were infected with 100 CFU *M. tuberculosis* expressing green fluorescence protein (GFP). 28 days later flow cytometry analysis was used to investigate intracellular niches of live and growing bacteria in lung immune cells. The proportion of cells that were positive for GFP: CD11b+Ly6G+ neutrophils, Siglec-F+CD11c+ autofluorescence+ alveolar macrophages, autofluorescence+Ly6Glo CD11b+Ly6C+/-monocytes and macrophages, B220+ B cells, autofluorescence-Ly6G-B220-MHCII+CD11c+ dendritic cells divided into either CD103+ or CD11b+ populations. Positive gates were drawn with the aid of cells derived from animals infected with *M. tuberculosis* without GFP. As the data were not normally distributed, differences between WT or *fcgrt-/-* were found using Mann-Whitney ranking, **p<0.01. Graph shows the median and interquartile range of cumulative data from 2 experiments. Data represent values from individual animals from 2 pooled experiments where n =4-5.
